# IHC Color Histograms for Unsupervised Ki67 Proliferation Index Calculation

**DOI:** 10.3389/fbioe.2019.00226

**Published:** 2019-10-01

**Authors:** Rokshana S. Geread, Peter Morreale, Robert D. Dony, Emily Brouwer, Geoffrey A. Wood, Dimitrios Androutsos, April Khademi

**Affiliations:** ^1^Image Analysis in Medicine Lab, Ryerson University, Toronto, ON, Canada; ^2^School of Engineering, University of Guelph, Guelph, ON, Canada; ^3^Ontario Veterinarian College, University of Guelph, Guelph, ON, Canada

**Keywords:** breast cancer, color deconvolution, color image processing, hematoxylin, Ki67, color separation, histogram, proliferation index

## Abstract

Automated image analysis tools for Ki67 breast cancer digital pathology images would have significant value if integrated into diagnostic pathology workflows. Such tools would reduce the workload of pathologists, while improving efficiency, and accuracy. Developing tools that are robust and reliable to multicentre data is challenging, however, differences in staining protocols, digitization equipment, staining compounds, and slide preparation can create variabilities in image quality and color across digital pathology datasets. In this work, a novel unsupervised color separation framework based on the IHC color histogram (IHCCH) is proposed for the robust analysis of Ki67 and hematoxylin stained images in multicentre datasets. An “overstaining” threshold is implemented to adjust for background overstaining, and an automated nuclei radius estimator is designed to improve nuclei detection. Proliferation index and F1 scores were compared between the proposed method and manually labeled ground truth data for 30 TMA cores that have ground truths for Ki67+ and Ki67− nuclei. The method accurately quantified the PI over the dataset, with an average proliferation index difference of 3.25%. To ensure the method generalizes to new, diverse datasets, 50 Ki67 TMAs from the Protein Atlas were used to test the validated approach. As the ground truth for this dataset is PI ranges, the automated result was compared to the PI range. The proposed method correctly classified 74 out of 80 TMA images, resulting in a 92.5% accuracy. In addition to these validations experiments, performance was compared to two color-deconvolution based methods, and to six machine learning classifiers. In all cases, the proposed work maintained more consistent (reproducible) results, and higher PI quantification accuracy.

## Introduction

Breast cancer is the most common form of cancer among women (DeSantis et al., 2014) and further research and improved therapies are needed to reduce mortality rates. As pathology plays one of the most critical roles for patient management and diagnosis of breast cancer disease, there are opportunities to use this modality to improve patient care. Hematoxylin and eosin (H&E) slides have traditionally been used for this in the past, since these stains highlight cellular morphology and tissue microstructure. However, immunohistochemical (IHC) biomarkers have been gaining momentum in treatment planning, prognostication and research (Sargent et al., 2005), as they highlight the presence of proteins and the corresponding concentrations, which characterizes tumor behavior and activity. Currently, clinical IHC-based biomarkers such as Her2, ER, and PR are used to select adjuvant and hormone therapies for patients, which have resulted in improved patient care and outcomes (Hammond et al., 2010; Gosho et al., 2012; Guerrero-Zotano and Arteaga, 2017). Therefore, IHC slides have great potential to improve the quality of care for breast cancer patients.

In the last several years, Ki67 has been investigated as a clinical marker for breast cancer tumor aggressiveness and proliferation, exhibiting potential in predicting disease survival, recurrence, and response to various treatment options (Veronese et al., 1993; Jalava et al., 2006; Dowsett et al., 2011). The Ki67 biomarker, also known as MKI67, is a nuclear protein associated with cell proliferation (Schonk et al., 1989). To visualize cells that are in the process of dividing, tissue slides stained with diaminobenzidine (DAB) and counterstained with hematoxylin (H). Pathologists use scoring systems to estimate a proliferation index (PI); the number of positively stained tumor nuclei divided by the total number of nuclei in a specific region (Veronese et al., 1993). A higher PI indicates many cells are undergoing cell division, which can signify a more aggressive tumor.

Despite the utility of the Ki67 biomarker, there are challenges of incorporating it into clinical and research workflows. Manual PI measurement remains time consuming, and sensitive to inter- and intra-observer variability among pathologists (Dowsett et al., 2011; Gudlaugsson et al., 2012; Rizzardi et al., 2012; Shui et al., 2015). There have been international standardization efforts to reduce observer variability in PI measurement, but many challenges remain (Khademi, 2013). Points of discussion include variability in manually counted cells (Khademi, 2013), the selection of appropriate cut-off thresholds for protein detection (Albarracin and Dhamne, 2014), the number of high power fields to evaluate (Harris et al., 2016), and the PI ranges that correlate to prognosis, i.e., low (<10%), intermediate (11–30%), and high (>30%) levels of proliferation activity (Khademi, 2013). The European Society for Medical Oncology (ESMO) and the American Society of Clinical Oncology have concluded that Ki67 would be a useful clinical tool if it was standardized (Dowsett et al., 2011; Senkus et al., 2015). Therefore, to increase clinical utility and adoption of Ki67, robust standards development, research and tools to improve the consistency of PI quantification are needed.

Automated image analysis and machine learning tools for digital pathology, broadly known as computational pathology, are a great solution to combat these challenges. They offer efficient, reliable and objective PI measurement for large amounts of data. There are reimbursement models in the US that support using digital image analysis to improve consistency of IHC analysis for clinical use (Zarella et al., 2019). Such tools can be used to assist pathologists in obtaining reproducible PI measures (Luporsi et al., 2012), develop more consistent Ki67 scoring guidelines as well as for breast cancer research.

Commercial algorithms for Ki67 have shown they are capable of attaining fast and objective PI estimates (Pantanowitz, 2010; Rohde et al., 2014), but because of the variability in Ki67 images it is an on-going research topic to improve performance. In Acs et al. (2019), the authors compared the reproducibility of Ki67 PI estimates of three well-known Digital Image Analysis (DIA) platforms: HALO, QuantCenter and QuPath. Each method is based on color deconvolution, cell segmentation and is followed by machine learning to calculate the PI. In one experiment, each algorithm was trained using the same slide at two different times, separated by 4 days. The generated models were then used on the same test set and there were differences in the generated PI estimates. Although the differences were small, the values were found to be different, which highlights the importance of training data, and reproducibility challenges for machine-learning algorithms. In Koopman et al. (2018) the authors compared PI accuracy of two commercial products from Visiopharm and HALO. Using 20 breast carcinoma cases for training, 154 breast carcinomas specimens for testing, the spearman's correlation coefficient between manual and automatic PI values ranged between 0.93 and 0.94.

Research works in the literature have explored novel methods for automated Ki67 PI calculation. In Polat and Güneş (2007) a least-squares support vector machine with nine cellular features was constructed to automatically classify regions of interests from 683 samples of human breast tissues from a single center (Wisconsin Breast Cancer Dataset). Using 10-fold cross validation with 50–50 training and test splits, the highest accuracy of 98% was reported. In Kårsnäs et al. (2011) an iterative dictionary learning approach was used for classification of tissue into probability maps of each stain type. The learning algorithm automatically updated the dictionary using the training images for final implementation on the test images. A 7.7% error rate in PI measurement was found, on a dataset of 58 TMAs.

Deep learning methods are gaining momentum in medical image analysis and have been applied to Ki67 quantification as well. In Saha et al. (2017), a convolutional neural network (CNN) was used to classify detected nuclei as immunopositive or immunonegative. An F-score of 0.91% was reported. It was stated that a 70–30 split was used for training and validation, and in total, 450 regions of interest were taken from 90 slides, from a single center. In Zhang et al. (2018), deep learning was implemented to detect tumor regions in Ki67 images. The proposed method used a combination of two models; CNN for image classification and a Single Shot Multibox Detector (SSD) for object detection. The CNN and SSD model obtained an accuracy of 98 and 90%, respectively, and Ki-67 quantification results were not reported. The overall proposed method utilized a total of 3000 tiny image patches, which are complemented by data generated by an adversarial network (GAN). The details on the number of patients the patches come from, as well as the imaging center(s) is unknown.

Although supervised learning methods are lucrative, there can be challenges for medical image analysis. For state-of-the-art methods, very large datasets with annotations are required to achieve good performance. Generating large enough, representative annotated datasets for training a Ki67 model is a challenge, as they are laborious and time consuming to create, as well as subject to observer variability. Additionally there are sources of noise and variability in Ki67 images that create challenges for all automated approaches. For example:

1. **Multi-Institutional Data:** Across labs and institutions, there are different scanning hardware systems and stain compounds resulting in color variability across images, tissues, biomarkers and patients (Krishnamurthy et al., 2013).
2. **Slide Preparation:** Different slide preparation and staining protocols can create stain inconsistency, overstaining, and other image artifacts. DAB overstaining causes stromal regions and biomarker negative nuclei to carry DAB stain, which can create false positives and negatives.
3. **Biomarker Levels:** Biomarker expression level varies from patient to patient, and even within tumors due to disease heterogeneity. There can be wide variability in the intensities and colors for biomarker positive nuclei. Low biomarker concentrations translate into lightly stained Ki67 regions, whereas high Ki67 concentrations are darkly stained regions.
4. **Ki67 Detection:** The Ki67 positivity threshold (protein detection cut off threshold) can be difficult to determine, especially in the presence of overstaining.
5. **Nuclei Size:** Nuclei can be of varying size depending on the magnification level (resolution) of the images as well as tumor cells, creating objects at a variety of scales to be detection.

To enable clinical-use and large-scale studies of Ki67, automated PI quantification tools must provide robust and consistent results across all images and patients. For clinical implementation, ideally, algorithms should be vendor-agnostic, efficient and robust to data variability, so they can be deployed in any clinical center and lab. For Ki67 standards development and research, algorithms must analyze multi-center, international datasets robustly and reliably.

To overcome these challenges, in this work, we investigate a fully automatic, unsupervised method for Ki67 PI quantification that is robust on multi-center, highly variable data. It is unsupervised and depends on a novel color separation method that discriminates between DAB and H stains using the IHCCH, which can separate brown and blue colors robustly, regardless of the variation in color, or biomarker expression level. It can model both lightly and darkly stained images within one framework and an adaptive threshold that suppresses background overstaining. A novel adaptive nuclei segmentation method is also developed to capture cells of different sizes.

The algorithm is first applied to 30 canine mammary tumor tissue microarray (TMA) core images and performance was evaluated against 129,404 manually labeled ground truths for Ki67+ and Ki67− cells. Performance is measured in terms of nuclei detection and PI quantification accuracy. The proposed work was then applied to 50 human breast tissue TMA images from Protein Atlas database (Uhlen et al., 2017). These two datasets, from two different international institutions, have variable PI scores and biomarker expression levels, background staining, and color variability, representing a challenging dataset to quantify. Performance is compared to six machine learning classification methods and two color deconvolution approaches: ImageJ (Schneider et al., 2012), and Immuno-Ratio (Tuominen et al., 2010). Lastly, through visual examples, it is demonstrated how the novel color separation method based on the IHCCH can be easily applied to other biomarkers, such as ER, PR, and Her2, which provides a robust, new way to detect stains in IHC images.

## Methods and Materials

For robust and accurate automated PI estimation algorithms, and IHC analysis in general, separating the hematoxylin (H) stained nuclei from the IHC positive structures is the first and most critical piece of the pipeline. Such a tool allows for the biomarker positive regions to be analyzed separately from the negative regions, which improves robustness. Inconsistencies in this phase can greatly affect successive nuclei detection and PI quantification.

Color deconvolution (CD) proposed in Ruifrok and Johnston (2001) has dominated digital pathology image analysis as a pre-processing step for automated stain separation. It has been applied to Ki67 analysis for stain deconvolution and PI estimation (Kårsnäs et al., 2011; Shi et al., 2016; Mungle et al., 2017). CD is dependent on the Beer-Lambert (BL) law of absorption (Ruifrok and Johnston, 2001; Macenko et al., 2009) which characterizes each pure stain by an optical density (OD) vector of light in the red, green, and blue (RGB) intensity channels (Kårsnäs et al., 2011). The OD space linearizes the relationship between stain concentrations (Di Cataldo et al., 2010) and the stain vectors are used to un-mix the stain concentrations on a per pixel basis.

**Figure 1:**
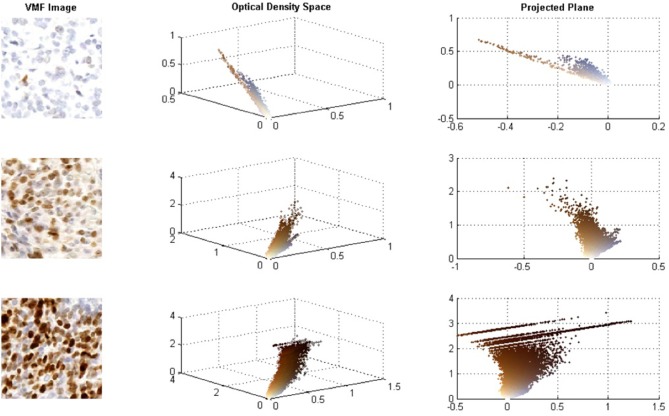
**(A)** Sample images of low, intermediate, and high Ki67 stain concentrations. **(B)** RGB optical density space for respective images. **(C)** Projected plane obtained via principle component analysis on the RGB OD color space.

Although CD has been used for IHC analysis with promising results, for high concentrations of Ki67, the DAB stain is dark and creates non-linear properties in the BL Law (Konsti et al., 2011). For darkly stained regions, DAB stains are not true absorbers of light and there is light scattering, which does not follow the BL law of light absorption (Macenko et al., 2009; Varghese et al., 2014). Figure 1 demonstrates how DAB stained images behave in the OD space. The images on the left contain the original Ki67 images, the middle images contain the 3D scatter plot of the OD RGB values (color-coded), and the right contains the 3D scatter plot projected on the plane of best fit. At low DAB stain concentration levels, DAB and H are linearly separable in the OD space and BL is effective to separate hematoxylin, and DAB stains in this case. However, for higher Ki67 concentrations, light scattering creates non-linearities in the OD space—brown pixels are spread over a larger region in a non-linear fashion and there are significant quantization effects at the darkest stain levels. In these cases, the BL law may not be as effective in separating hematoxylin and Ki67 stains and errors in the estimated stain concentration images can occur.

In addition to these challenges, even for lightly stained images, implementation of CD is also dependent on the selection of stain vectors to generate an OD deconvolution matrix. In Ruifrok and Johnston (2001), the authors implemented CD with predefined stain vectors for DAB and hematoxylin for stain separation but found the resultant stain concentration images to have missing information in both the DAB and hematoxylin channels. Other methods (Taylor and Levenson, 2006; Shi et al., 2016) have recognized CD results vary for different staining and imaging protocols and have focused on adaptively defined stain vectors on a per image basis (Taylor and Levenson, 2006). However, due to the non-linearity properties of the BL law for darker stains, and variability of IHC images in general, adaptive estimation of stain vectors is challenging and can be inaccurate for automated stain separation and PI calculation.

**Figure 2:**
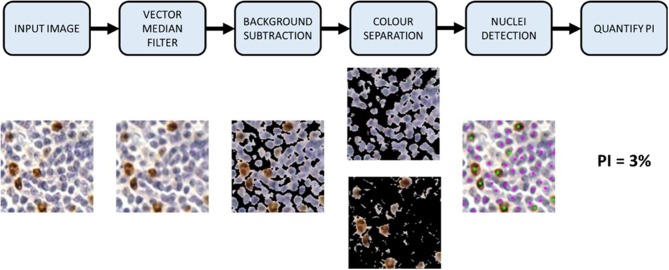
Proposed automated Ki67 proliferation index calculator framework.

In this work, we propose a novel color separation method that discriminates between DAB and H stains using the IHCCH, which does not dependent on the BL and can separate brown and blue colors robustly, regardless of the variation in color, or biomarker expression level. It and can model both lightly and darkly stained images within one framework. The complete Ki67 PI quantification pipeline is shown in Figure 2. First, the TMAs are preprocessed with a color vector filter and background subtraction, followed by color separation. An adaptive threshold is used to adjust for DAB overstaining and separate DAB and H channels robustly. An automated nuclei size estimate is used to detect and count cells from both DAB and H channels. The PI is computed based on the number of Ki67+ and Ki67− nuclei, which is compared to the pathologists' ground truths to determine performance. This section will further detail the methods, validation metrics, and data used.

### Preprocessing

Due to the noise and variability in intensities/color in histopathology images, a de-noising procedure is necessary. Histopathology images consist of color (RGB) images and the application of a scalar de-noising filter to each channel individually may result in the loss of correlation between channels and the skewing of colors (Macenko et al., 2009). Instead, a Vector Median Filter (Plataniotis and Venetsanopoulos, 2013) was applied to the images to make colors appear more uniform in the images by preserving color proportions and content. The Vector Median Filter calculates the median color vector within a defined pixel neighborhood, using a 3 × 3 window, for every pixel in the image that contains non-zero values. By treating the pixels as vectors instead of scalars, the resultant filtered image retains the relationship between color channels and edges can be maintained better than the scalar counterpart, while smoothing in low frequency regions. After de-noising, a second preprocessing step known as background subtraction was performed to isolate tissue and cell regions and suppress background regions. A local mean adaptive threshold was applied to each image in the grayscale luminance channel because both DAB and H stained pixels are darker than their surroundings allowing application of a single threshold to capture both stains. A local mean adaptive method is an iterative algorithm that measures a local threshold determined by the mean grayscale intensities of a neighborhood. This method was preferred over other popular methods such as Otsu's method (Otsu, 1979) because it is local, and can adapt to different stain and tissue proportions.

### Color Separation

In this section, it will be shown that the b^*^channel of the L^*^a^*^b^*^ color space is an efficient color representation that can be used to automatically separate blue and brown colors for effective DAB and H stain separation. IHC analysis performed by a pathologist relies on the human visual system's (HVS) perception of staining intensity and color characteristics and the L^*^a^*^b^*^ space is a perceptually linear color space that mimics the perception of color achieved by the human eye (Tkalcic and Tasic, 2003). The L^*^a^*^b^*^ color space is derived from XYZ tristimulus values (Brey et al., 2003), which models the HVS non-linear response to color perception with a cubic root relation. Chromatic information is decoupled effectively from intensity allowing automated approaches to examine purely color content, regardless of its perceived luminance. The L^*^ channel represents luminance of an image, while the a^*^ and b^*^ axes represents complementary chromatic content. Specifically, the a^*^ channel differentiates complementary red and green hues while the b^*^ channel differentiates complementary blue and yellow hues.

Color separation of hematoxylin (blue) and Ki67 (brown) is achieved through a method that differentiates colors associated with each stain by focusing on the complementary yellow and blue characteristics within the chrominance channel of IHC images. The negative values represent “pure” or saturated blue hues, zero represents no color content, and the positive region defines “pure” or saturated yellow hues. Colors of interest in Ki67 images are blue (H) and brown (DAB), where brown as a color is composed of dominant yellow or orange hues (red + green) mixed with black or blue in lower proportions (Cook et al., 2005). Since brown is dominated by red and green components and blue colors associated with H are dominated by the blue hues, the channel has the potential to discriminate between DAB and H stains.

**Figure 3:**
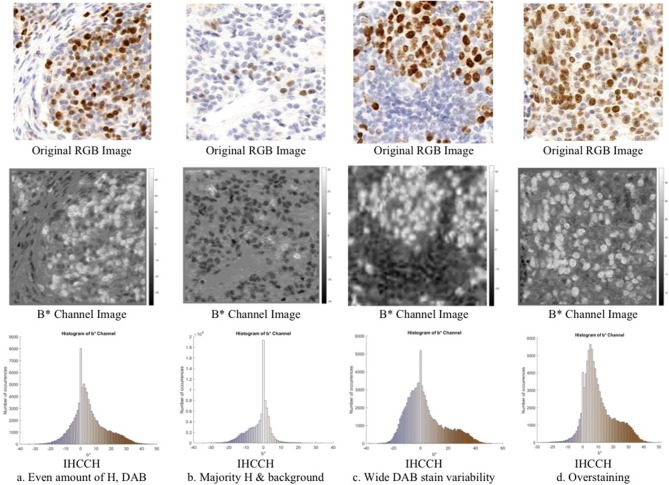
Region of interest from OVC dataset of Ki67 TMAs (**top**) with corresponding b^*^channel images (**middle**) and IHCCH plots (**bottom**).

Consider Figure 3 (middle row), which contains an RGB Ki67 ROI, and the corresponding b^*^ channel image *b*(*x, y*), where (*x, y*) ϵ Z^2^ are the spatial coordinates. In *b*(*x, y*), the brown regions (DAB) are associated with high intensities (positive b^*^ values), while the blue stain (H) is associated with low intensities (negative b^*^ values). To explore this relationship further, a novel histogram representation of the b^*^ channel, called the IHCCH, is presented in Figure 3 (bottom row). The bins of the histogram are color coded according to the average RGB color at that particular b^*^ value. Blue hues from the H stain are located in the negative b^*^ region of the histogram and as the color saturation decreases (lower H concentration) the b^*^ values approach zero. This is even true for light purple or other blue-ish hues. For DAB staining, a similar effect is observed as positive b^*^ values represent the relative saturation of yellow (red and green) hues, which are the dominant color components in brown. Light browns are close to the origin (low R and G values), and darker browns correspond to larger values of b^*^ (higher R and G values). Light and dark Ki67 brown colors are consistently described by positive b^*^ values regardless of the intensity and darkness. For regions with large amounts of overstaining (Figure 3D), the overstained regions (light brown in color), are represented by a high number of occurrences in the first few bins of the positive b^*^ values. This provides a robust mechanism to manage overstaining, as low values of b^*^ can be suppressed.

In order to separate IHC images into H and DAB components, two thresholds computed from the IHCCH are used to threshold for color separation into blue and brown. The first threshold, *T*_*blue*_, is used to globally threshold and isolate H pixels, and the second threshold denoted *T*_*brown*_ is used to find DAB pixels. The b^*^channel *b*(*x,y*) is then scaled to create images and which are confidence images ∈ [0,1] that describe the level of saturation of blue and brown for H and DAB stains, respectively. A value of 0 is associated with little or no stain color content (achromatic) and a value of 1 represents the strongest or most saturated stain color content for each stain. By scaling the confidence images to the minimum and maximum observed b^*^ values within each image, the contrast between low and high staining is increased, and eases detection of H and Ki67+ regions. Formally, *H*(*x, y*) and *D*(*x, y*) are found by thresholding and scaling, as in

(1) $$\begin{array}{l} H\left(x,y\right)=\left\{\begin{array}{c} \frac{-b\left(x,y\right)\;+\;T_{blue}\;}{b_{\min}}\text{ for }b\left(x,y\right)\;<\;T_{blue}\\ \;0\text{ for }b\left(x,y\right)\;\geq \;T_{blue} \end{array}\right\} \end{array}$$

(2) $$\begin{array}{l} D\left(x,y\right)=\left\{\begin{array}{c} \frac{b\left(x,y\right)\;+\;T_{brown}\;}{b_{\max}}\text{ for }b\left(x,y\right)\;>\;T_{brown}\\ \;0\text{ for }b\left(x,y\right)\;\leq \;T_{brown} \end{array}\right\} \end{array}$$

where *b*(*x, y*) is the b^*^ channel image at pixel locations (*x, y*), *b*_min_ is the minimum b^*^ intensity and *b*_max_ is the maximum b^*^ intensity in the image. The (*x, y*) locations where *H*(*x, y*) and *D*(*x, y*) are nonzero value [i.e. *H*(*x, y*) > 0 and *D*(*x, y*) > 0] are the detected regions that contain blue and brown colors, respectively, and are used to create binary masks for the DAB and H stains. The binary masks can be used to mask the R, G, and B channels to generate color images that represent the segmented blue and brown regions.

A natural choice for the thresholds would be *T*_*blue*_ = *T*_*brown*_ = 0, since b^*^ = 0 marks the absence of color. However, as seen in Figure 3, DAB overstaining results in large amounts of DAB background staining which falsely stains H nuclei. As a result, thresholds *T*_*blue*_ = *T*_*brown*_ = 0 could cause false positives in the *D*(*x, y*) channel due to background staining, or false negatives in the *H*(*x, y*) channel by missing lightly stained Ki67− nuclei.

To determine a threshold that robustly detects DAB positive and negative regions in the presence of background overstaining, an adaptive threshold is designed to mimic a pathologist's response to variably stained images. Pathologists typically “raise” their threshold for determining the Ki67+ regions in the presence of overstaining, which allows them to robustly detect Ki67 and H nuclei. Inspired by this, this behavior is modeled through an adaptive histogram thresholding using an iterative algorithm that searches for a threshold that optimally separates two classes based on the “weight” of the histogram on either side of the threshold. In the presence of overstaining, the histogram is heavy on the right side, and the threshold is iteratively increased to “balance” the histogram.

The adaptive threshold method is as follows. Utilizing the IHCCH, an initial threshold at b^*^ = 0 is defined. The “weight” of the histogram on either side of the threshold is computed and depending on the amount of staining content, the threshold is shifted left or right by one bin. For example, a large amount of DAB overstaining will result in the histogram being heavily weighted on the right side and consequently, the threshold will be shifted to the right. This process is iterated until the threshold is found that “balances” the histogram on either side of the threshold.

Formally, for the ith iteration with, *T*_*bi*_*n*__*i*__ being the currently selected threshold, first, the sum of the histogram counts on either side of the threshold are found by:

(3) $$\begin{array}{l} A_{i}=\sum_{b=T_{bin_{i}}}^{N_{i}}h\left(b\right)\;,\;b\;=\;\left[T_{bin_{i},}\;T_{bin_{i}}+\;1\;,\;\ldots \;,\;N_{i}\right] \end{array}$$

(4) $$\begin{array}{l} B_{i}=\sum_{b=M_{i}}^{T_{bin_{i}}}\;h\left(b\right)\;,\;b\;=\;\left[M_{i},\;M_{i}+\;1\;,\;\ldots \;,\;T_{bin_{i}}\right]\; \end{array}$$

where, h(b) is the b^*^ histogram (IHCCH), *b* is the b^*^ value, *A*_*i*_ and *B*_*i*_ are the sum of histogram counts on either side of the threshold *T*_*bin*_. *N*_*i*_ and *M*_*i*_ determine the intervals used to compute *A*_*i*_ and *B*_*i*_. If *A*_*i*_ > *B*_*i*_, the histogram is heavy on the right side, and the threshold is moved to the right by one with *T*_*bin*_ = *T*_*bin*_ + 1 and a bin is removed *M*_*i*_ = *M*_*i*_ + 1. If *A*_*i*_ < *B*_*i*_, the threshold is shifted to the left by one with *T*_*bin*_ = *T*_*bin*_ − 1 and *N*_*i*_ = *N*_*i*_ − 1. A final optimized threshold T^*^ is determined when the change in the threshold is minimized between iterations and $T_{blue}=T_{brown}=T^{*}$. In DAB overstained images, the histogram will be heavy on the right and the threshold will be successively shifted to the right until there is a balance.

### Nuclei Detection

To count nuclei and determine a proliferation index, a nuclei detection algorithm was implemented to quantify the number of individual nuclei in the separated hematoxylin and Ki67 color channels. A modified version of the nuclei detection algorithm proposed in Qi et al. (2012) is utilized due to its ability to identify overlapping and clustered nuclei. The method was originally proposed for the grayscale stain channels of H&E images, however, in this work, we use the color-separated DAB *D*(*x, y*) and *H*(*x, y*) images for nuclei detection. It depends on the gradient magnitude and direction, along with a cell radius parameter to estimate nuclei centers. A voting image is used to determine the center of a cone area with the supplied cell radius. The mean shift algorithm is used to determine final nuclei seed locations.

In Qi's original work, a cell radius parameter *r* was supplied by the user to detect nuclei. Instead, we introduce an adaptive cell radius estimator to automatically determine the cell radius parameter *r*. This eliminates user interaction and ensures the framework provides repeatable and reliable results, regardless of the magnification factor or size of the cells. The cell radius estimate is determined automatically by: (1) creating a binary foreground mask for each stain [*H*(*x, y*) > 0 and *D*(*x, y*) > 0], (2) estimating the area of unique objects, (3) generating a sorted histogram of object areas, (4) trimming the top and bottom 20% of objects according to their area, (5) estimating cell radius on the remaining objects. Trimming the histogram of object areas removes really small or large objects, with cellular objects remaining. The cell radius is then by approximating the area of the remaining objects with the equation of a circle A = πr^2^. Since cancerous nuclei (DAB stained) are usually larger compared to Ki67− nuclei (H stains), the average nuclei radius is estimated separately for each channel.

### Validation Metrics

To validate the proposed methodology, two types of validation tests are performed that measure nuclei detection performance and PI measurement accuracy. Nuclei detection performance was assessed through calculation of an F1 score, which incorporates both sensitivity and precision to give an overall measure of algorithm performance and is found by:

(5) $$\begin{array}{l} \text{F1}=\;\frac{2\;\times \;TP}{\left(2\;\times \;TP\;+\;FP\;+\;FN\right)} \end{array}$$

where TP is true positive, FP is false positive, and FN is false negative. A circular validation window was examined for each automatic seed location resulting in a true positive if the window contained a coincident seed from the manually labeled data and a false positive if a labeled seed was absent. Once a manual seed was paired with an automatic seed it was removed from the manual data to ensure no repeated counting of a labeled seed occurred. The manual seeds unaccounted for after validation represent the false negatives not detected by the proposed method.

To assess the PI accuracy, first, the PI is computed by for an entire TMA by:

(6) $$\begin{array}{l} \text{PI}=\;\frac{\#\;Positive\;Nuclei\;}{Total\;\#\;Nuclei}\times 100\% \end{array}$$

Manual and automatic PI estimates are compared through correlation analysis, PI differences, intra-class correlation coefficient (ICC) calculations and Bland Altman plot comparison.

### Experimental Data

**Figure 4:**
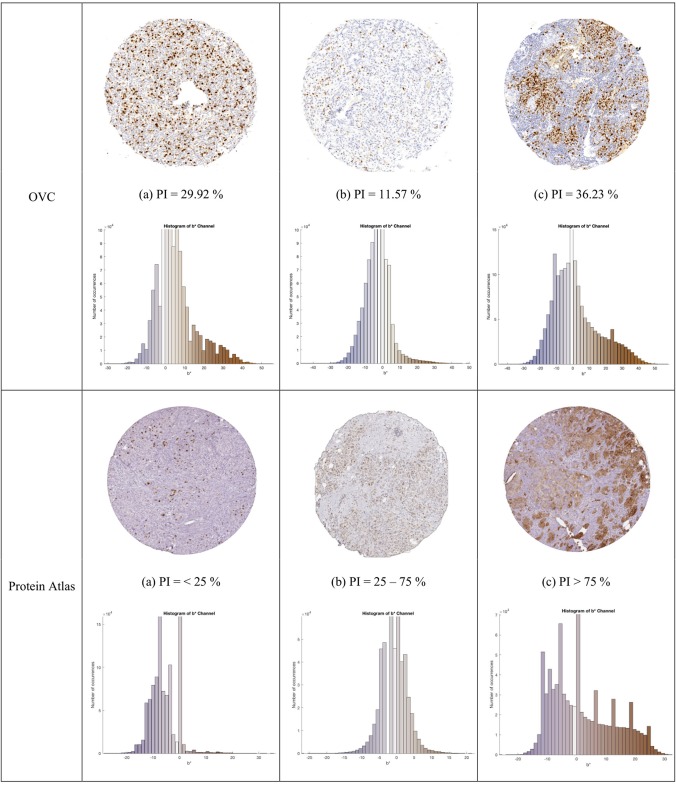
TMA images from OVC (**top**) and Protein Atlas (**middle**) datasets, with corresponding IHCCH below.

**Figure 5:**
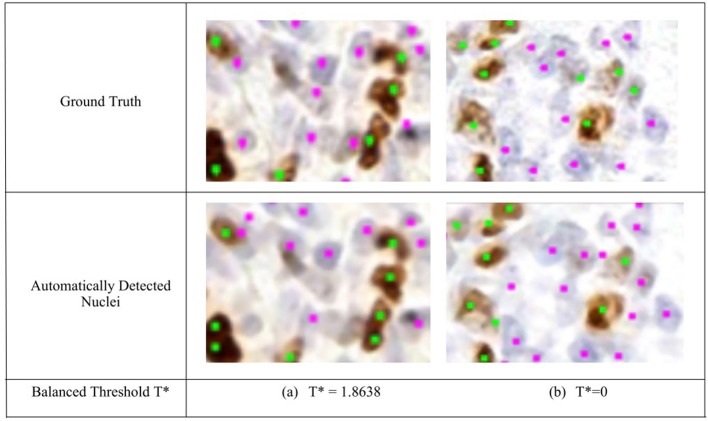
Region of interest from OVC Ki67 TMA. Nuclei detection for H and DAB channels, for automated and manual approaches. (**Top**) Ground truth annotations. (**Bottom**) Automatically detected nuclei using the balanced threshold method. Green marker is for Ki67+ and Pink for Ki67−.

A total of 30 canine mammary TMA core images with 129,404 individually labeled nuclei was obtained from the Ontario Veterinary College (OVC) at the University of Guelph. The TMAs were scanned with a Leica SCN400 Slide Scanner at 20X magnification, image resolution of 72 DPI and pixel spacing of 0.5 μm. Individual TMA core images were cropped from whole slide images using Pathcore's Sedeen (Martel et al., 2017). Manual counting was performed in ImageJ (Schneider et al., 2012) by zooming in on the image and placing seed markers at Ki67+ and Ki67− nuclei locations using a computer mouse. This was repeated until the entire core was labeled. Three example TMAs, exhibiting varying biomarker expression levels, PI and color variability are shown in Figure 4. An example region of interest, along with the annotations from the OVC dataset is shown in Figure 5.

Another dataset containing 50 Ki67 TMA core images from the Protein Atlas (Uhlen et al., 2017) was used to further assess the proposed approach. This open source dataset, has Ki67 protein expression data acquired from an IHC lab in Uppsala, Sweden. The TMAs were digitized using Aperio ScanScope® AT or Aperio ScanScope® T2, with an image resolution of 96 DPI. The TMA's were scored according to three PI ranges: < 25, 25–75, and >75%. Three example TMAs from this dataset are shown in the bottom row of Figure 4.

The combined dataset containing 80 multi-institutional TMA images scanned using three different scanners, two different continental processing laboratories with contrasting staining protocols, results in a broad diversity of patients, stain vendors, color variation, image resolution, and variable staining levels. The IHCCH plot of each corresponding TMA can be seen below each TMA in Figure 4. There is wide range of stain variation, color variability, and varying levels of biomarker expression.

## Results

In this section, the experimental results will be presented. First, the visual results of the IHCCH method will be shown, followed by quantitative results for the automated radii estimator and thresholding method to separate the DAB and H channels. The OVC data is used for this, since this dataset contains individually annotated nuclei. Using the optimized/validated algorithm, the next subsection contains the performance on the Protein Atlas dataset. Since the Protein Atlas data's ground truths are PI ranges, the accuracy was measured based on a binary quantification system, i.e., whether or not the automatically generated PI value falls within the ground truth range. The final sections of results compare the proposed method to two color devolution frameworks and six supervised learning models. Both the OVC and Protein Atlas datasets are used for these experiments.

### Visual Analysis

**Figure 6:**
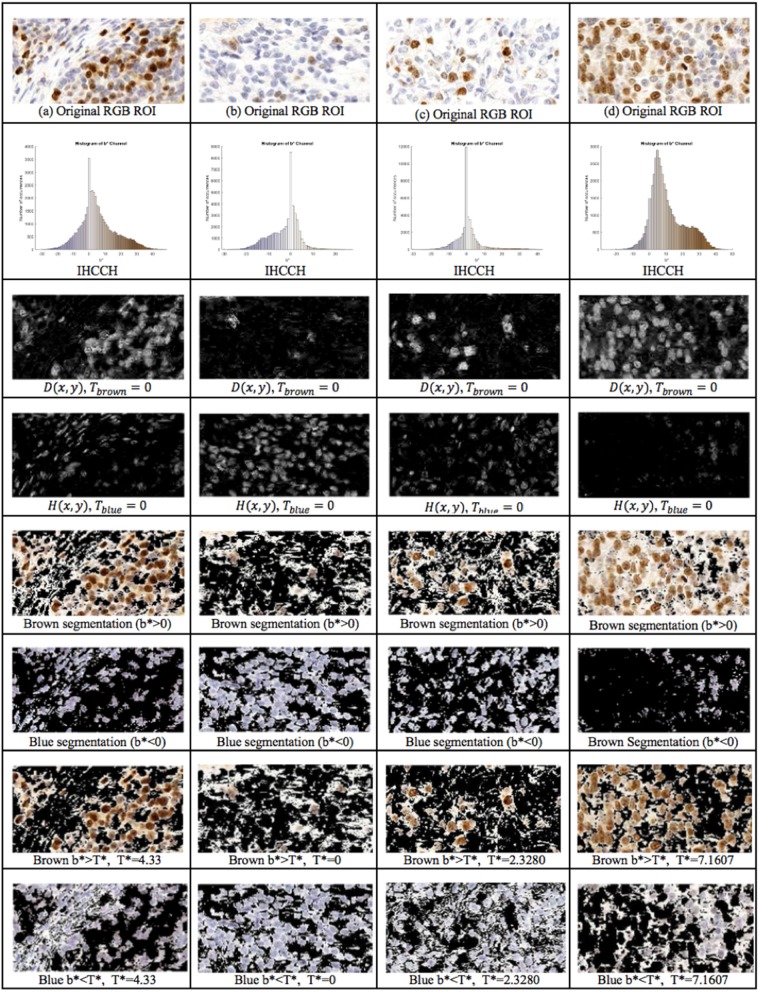
1st Row: Region of interest from Ki67 TMA^.^ 2nd Row: IHCCH, 3rd and 4th row: DAB and H confidence maps for *T*_*blue*_ = *T*_*brown*_ = 0. 5th Row: Brown region (DAB) segmentation with *T*_*brown*_ = 0. 6th Row: Blue region (H) segmentation with *T*_*blue*_ = 0. 7th Row: DAB segmentation using adaptive threshold $T_{brown}=\;T^{*}$. 8th Row: H segmentation using adaptive threshold $T_{blue}=T^{*}$.

The proposed framework introduces the IHCCH and thresholding to determine DAB and H stains. Using a threshold of *T*_*blue*_ = *T*_*brown*_ = 0 (i.e., b^*^ = 0), example segmentation results for four regions of interest are shown in Figure 6. The top row contains the original Ki67 image, the second row contains the IHCCH and the third and fourth rows show the confidence images *D*(*x, y*) and *H*(*x, y*). The fifth and sixth rows show the resultant blue and brown segmentations generated by segmenting the confidence images. Blue and brown regions are robustly detected for a variety of color variations and staining intensities. However, for images with DAB overstaining (a,d), the brown segmentation contains both overstained Ki67− nuclei and background staining. In the blue region, negative nuclei that absorbed DAB stain have not been detected. Inaccuracies in stain detection can impede nuclei detection.

To remove background DAB overstaining, the brown region is segmented by the adaptive threshold T^*^ obtained by the balanced histogram approach as shown in the seventh row of Figure 6. In the DAB channel, background overstaining regions have been suppressed in the new segmentation result, and Ki67− nuclei have been excluded. Regions of interest with larger amounts of background DAB staining generates a larger threshold thus removing some of the overstained regions. In Figure 6c, T^*^ was found to be <0 since there is low amounts of background DAB staining and H dominates the image. In this case, the threshold was constrained to be T^*^ = 0. The eighth row illustrates the balanced thresholding method of $T_{blue}=T_{brown}=T^{*}$, exemplifying that H nuclei are robustly detected, regardless of the level of overstaining. The gradient based nuclei detection method is used to detect nuclei in *D*(*x, y*) and *H*(*x, y*) to count Ki67+ and Ki67− nuclei, respectively, for automated PI calculation. Example nuclei detection results, alongside the annotations, are shown in Figure 5.

### Automated Radius Estimator and Optimal Threshold

The following section validates two vital components for the framework: cell radius estimator and IHCCH thresholding technique. Cell nuclei detection was evaluated across the OVC dataset for each stain type independently. Arbitrary radii estimates were selected to represent a range of possible values selected by a user and were then compared against the results obtained via the automatic cell radius estimator framework. Nuclei detection performance was assessed through calculation of an F1 score between the automated and manually detected nuclei from the DAB and H channel separately. Cell radii estimates were validated using a circular validation window with diameter equal to 2X the automatically determined radius.

**Figure 7:**
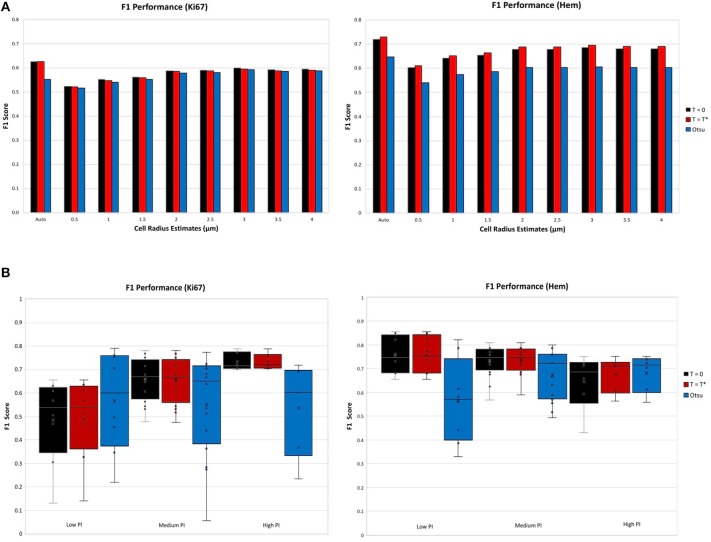
F1 scores for nuclei detection for each threshold, per stain. **(A)** F1 scores comparison of automatic cell radius estimator per thresholding condition. **(B)** F1 performance of automatic thresholding condition, in ranges of low, medium, and high PI.

The F1 score for various user defined cell radius estimates were observed for the achromatic boundary: *T*_*blue*_ = *T*_*brown*_ = 0, the “overstaining” threshold: $T_{blue}=T_{brown}=T^{*}$, which are both compared to Otsu's threshold a well-known histogram thresholding method (Otsu, 1979). Figure 7A (top) illustrates the F1 performance score of each threshold per stain. The F1 score performance for both Ki67 and hematoxylin are for the automatic cell radius estimated and eight possible user selected parameters ranging from 0.5 to 4 μm. For both stains, the balanced threshold ($T_{blue}=T_{brown}=T^{*}$) outperforms the achromatic and Otsu thresholds. With the “balanced” threshold, the user selected nuclei estimates yielded average TMA F1 scores of 0.67 and 0.57 for Ki67 and H, respectively, while the automatic cell radius estimate produced F1 scores of 0.73 and 0.63, respectively. The automatic cell radius estimator achieved a higher F1 performance score, affirming that the optimized balanced threshold with automatic radius estimation achieves optimal results. This avoids user input, which can be tedious, time consuming, and subjective. Automated cell radii estimator can also detect cells at different magnification levels.

Thresholds were evaluated using the OVC dataset for each stain type independently and performance was measured through the F1 score for the nuclei detected in H and DAB channels as shown in Figure 7B (bottom). Each threshold is examined according to ranges of PI: low (<10%), medium (10–30%) and high (>30%). Box plots are used to demonstrate the distribution and consistency of F1 score per threshold. Otsu's method achieved moderate F1 score for the Ki67 stain over all PI ranges, but has a large F1 variance, which reveals that the method is not reproducible or consistent in the Ki67+ stain. In the medium and high PI levels, the achromatic threshold (*T* = 0) and balanced threshold achieve similar F1 scores. For high PI values, the balanced threshold obtains a high F1 score with the lowest variation over the other methods, indicating reliability and consistency. Similar results are shown in the H channel. The optimized threshold, $T_{blue}=T_{brown}=T^{*}$, is optimal since it has highest and most consistent F1 performance.

**Figure 8:**
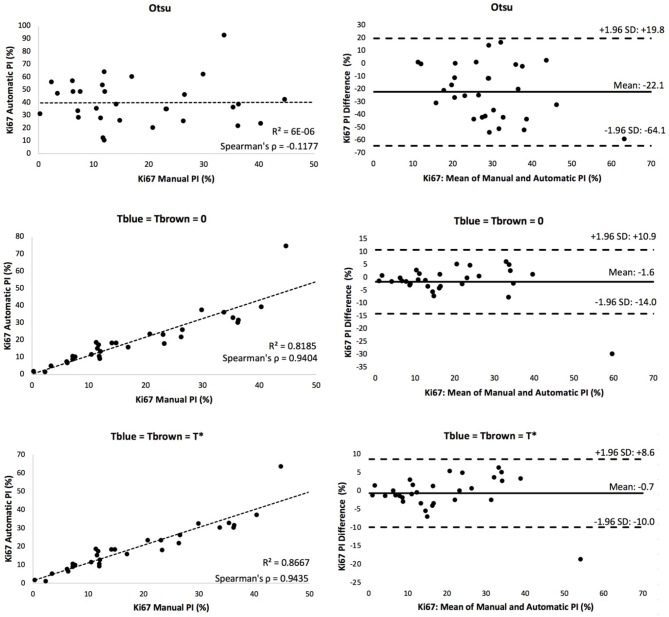
Scatter plot (**left**) and Bland Altman plot (**right**) of each experimental thresholding condition's PI results, with corresponding Spearman's coefficient.

A scatter plot of the agreement in PI between manual and automated approaches for each threshold is shown in Figure 8. The corresponding *R*^2^ and Spearman correlation were calculated, and the balanced histogram approach ($T_{blue}=T_{brown}=T^{*}$) achieved the highest Spearman's correlation coefficient of 0.944, with an *R*^2^ = 0.8667. This reveals a strong relationship between the automatic Ki67(+) PI estimator and the manually counted PI. The corresponding Bland-Altman plots based on the difference in PI between automated and manual approaches were computed and shown in Figure 8. The optimal threshold is the one with the lowest mean PI difference along with tight limits of agreements (LoA). Although the “natural boundary” (*T*_*blue*_ = *T*_*brown*_ = 0) have relatively low PI differences and LoAs, the balanced histogram threshold for overstaining achieves optimal results.

**Figure 9:**
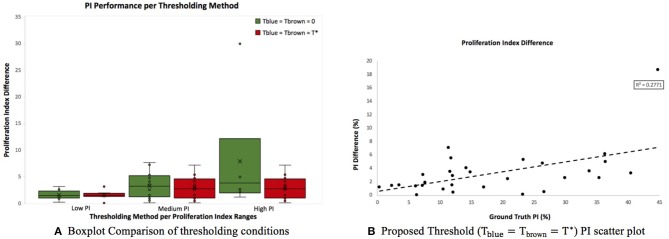
**(A)** Boxplot comparing PI difference performance between thresholds for low (<10%), intermediate (10–30%), and high (>30%) PI levels. **(B)** Correlation between PI difference and manual PI estimates for the balanced histogram approach and automated radii calculator.

Figure 9A is a boxplot of the difference between manual and automatic PI estimates of each threshold, with the corresponding levels of proliferation activity, i.e., low (<10%), intermediate (10–30%), and high (>30%) levels. Over all levels, the achromatic threshold obtained an average PI difference of 3.73% and the optimal balanced threshold (T^*^) for overstaining achieved an average difference of 3.3%. Otsu's thresholding method was excluded due to its low performance. The percentage error of the balanced histogram approach is lower for all three levels of proliferation activity levels, in comparison to the opposing methods. Despite slightly lower performance for the F1 score for Ki67+ nuclei detection, the PI estimates for the low PI range are still comparable to the manual ground truth and the variance is low too, indicating consistent and reliable performance.

Using the optimized threshold (T^*^) and the automated nuclei radius estimator on OVC data, the ground truth PI vs. the PI difference is plotted based in Figure 9B. As can be seen the error is below 8% except for one outlier. This further illustrates that the optimal thresholding method obtained accurate results in the presence of staining protocol variability, variable image resolutions, varied levels of biomarker expression, and scanner/stain color variation.

The intra-class correlation coefficient (ICC) (Fleiss and Cohen, 1973) for Ki67 PI quantification was lastly computed to quantify consistency and reproducibility of each method. The achromatic threshold achieved an ICC value of 0.943 and the optimized threshold for overstaining achieved the highest ICC value of 0.964. The overstaining threshold automatically produces an automatic PI with the closest correlation to the manually annotated PI values.

### Protein Atlas Data Validation

**Figure 10:**
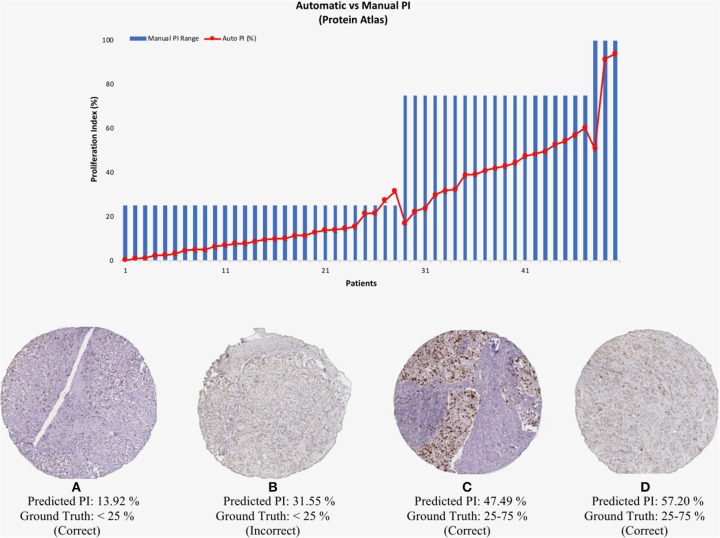
(**Top**) Automated PI results for Protein Atlas Dataset as a function of ground truth PI range. (**Bottom**) TMA core images from Protein Atlas with predicted PI values and corresponding ground truth ranges.

To test the validated method (balanced threshold with automated radii estimator), 50 Ki67 TMAs from Protein Atlas were used. The Protein Atlas dataset for Ki67 was manually annotated using a range, where each TMA was manually annotated with either low (< 25%), intermediate (25–75%) or high (>75%). The automated PI will be compared with the range of the ground truths. Figure 10 shows the PI estimate plotted as a function of the ground truth range. As can be seen the estimated PI falls into the range of the ground truth PI in many of the cases. Of the 50 TMA core images, five images were classified outside the given range, achieving an accuracy of 90% (45/50). Several example images are shown in Figure 10. Clearly, the proposed method is generalizing to new data.

### Comparison to Color Deconvolution Methods

Majority of quantification algorithms for digital pathology utilize CD for stain separation. As discussed, CD is not a reliable tool for color separation of DAB and H stains, since these stains do not abide by BL law of absorption for all stain levels.

To further investigate, the validated method is compared to the performance of two color deconvolution methods using the OVC dataset. In particular, the open source Immuno-Ratio Ki67 calculator (Tuominen et al., 2010) and ImageJ's CD method (Schneider et al., 2012) are used. ImmunoRatio uses color balancing, background subtraction and CD as preprocessing steps to obtain DAB and H images. The stain separated images are then postprocessed in order to segment nuclei, using methods such as particle filtering and the watershed algorithm. Finally, proliferation index is quantified, percentage of DAB-stained area over total nuclear area. For the ImageJ method, the built in color vectors for DAB and H stains were used to separate stains and each was saved as an individual image. The automated nuclei detector is then employed to detect nuclei in the DAB and H stains and calculate the PI.

Table 1 compares the average PI difference, linear correlation, and intra-class correlation coefficient of the other frameworks on the OVC data, compared with the performance of the balanced histogram threshold for overstaining. Table 1 shows that the ImageJ method has the largest mean PI estimate difference of 10.04%, while Immuno-Ratio has a mean PI difference of 4.65%, and the proposed method has the lowest PI difference of 3.25%. Similarly, the linear correlation, and ICC were higher for the proposed method. The proposed method acquired the highest linear correlation coefficient of 0.93 and the highest ICC value of 0.96, indicating a near perfect calculation of the PI values.

**Table 1:** Performance comparison to similar works.

	**ImageJ**	**Immuno-Ratio**	IHCCH (T_**blue**_ = T_**brown**_ = T*)
Mean PI difference	10.04	4.64	3.25
Linear correlation r	0.52	0.9	0.93
Intra-class correlation (ICC)	0.682	0.937	0.96

**Figure 11:**
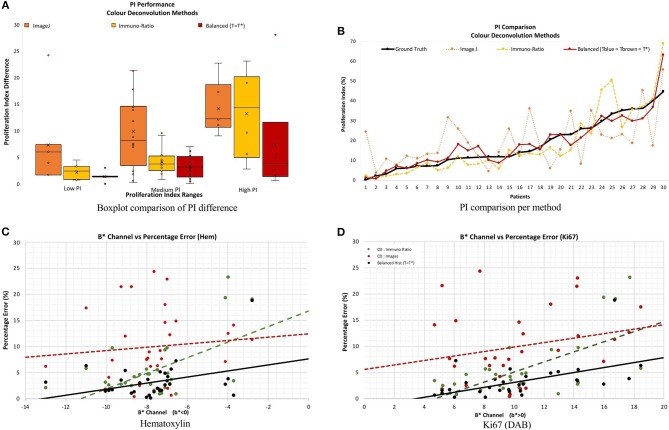
**(A,B)** PI difference and PI estimate comparison of all methods, including manual, ImageJ and Immuno-Ratio methods and balanced histogram (*T* = T^*^). **(C,D)** Percentage error in relation to the b* Channel within the L*a*b* Color space per stain, Hematoxylin **(A,C)** and Ki67 (+DAB) **(B,D)**.

Figure 11 serves as a visual representation of the issues related to utilizing CD as a stain separation method for proliferation quantification purposes. PI difference as a function of PI range, and estimated PI for the proposed and CD-based methods, is shown in Figure 11A. As the proliferation activity increases, the CD methods are less accurate probably due to stain variability, overstaining and breakdown of the Beer Lambert law. The balanced histogram method has the lowest variability and median when comparing PI differences. Figure 11B displays PI as a function of patient on the OVC data. Each line corresponds to the ground truth, or one of the methods being compared. Note that Figure 11B was sorted from lowest PI to highest based on the ground truth data, in order to visualize trends. The balanced histogram method is the closest in accuracy (most similar to ground truth line), in comparison to the CD algorithms which exhibit wide variation across the dataset, especially for higher PI values.

To examine results in relation to DAB's light scattering characteristics, Figures 11C,D contains the average percentage PI difference for each stain and method, plotted vs. the mean b^*^ value from the L^*^a^*^b^*^ color space. The mean value of the b^*^ channel, per image for each stain quantifies the dominating color in each of the channels. The background was excluded from the calculation of the mean b^*^ channel value, to achieve an objective comparison. Therefore, it is possible to examine algorithms' performances as a function of color, in an essence. Overall average b^*^ values, the proposed method achieves the lowest percentage error in PI estimation, and the slope of the line is low, indicating some consistency for a variety of colors. This is in contrast to the CD-based methods, such as the ImageJ CD method, which has large PI differences over all average b^*^ values, or the ImmunRatio method, which has a high slope indicating large variabilities for small or large average b^*^. Therefore, as b^*^ increases, the percentage error significantly increases in both CD methods which could be a result of the light scatter characteristics of the BL law in IHC. The balanced threshold has a consistent minimal percentage error in both stains, H and Ki67 (DAB), further illustrating the quantitative accuracy and robustness of the proposed method.

### Comparison to Machine Learning Classifiers

In this section, the performance of the unsupervised, proposed method, is compared to several supervised machine learning classifiers: Gaussian Naïve Bayes (NB), K-Nearest Neighbor (KNN), Logistic Regression (LR), Stochastic Gradient Descent (SGD), Support Vector (SVM), and Linear Support Vector (LSVC) Classifier. Both the OVC and Protein Atlas data were used for these experiments, and for consistency, the OVC data's PIs were converted into ranges to match that of the Protein Atlas, i.e., low (<25%), medium (25–75%), and high (>75%). These three labels are used as ground truth for training and testing. Therefore, in total, there are 80 TMA images with class labels that correspond the PI range. Classification accuracy is determined by the average classification performance over all three classes.

To compare the supervised and proposed unsupervised method fairly, the same preprocessing steps are completed for all implementations. Namely, the data is VMF filtered, background subtracted, and the color image is converted into a b^*^ channel image from the L^*^a^*^b^*^ space, and the histogram of the b^*^ channel is computed. The b^*^ histogram is used as the input to the machine learning algorithms to train and test the classifiers. In an essence, if the ML algorithms can correctly discriminate between classes, they are indirectly learning the optimal boundary between classes in this histogram to find the PI.

**Figure 12:**
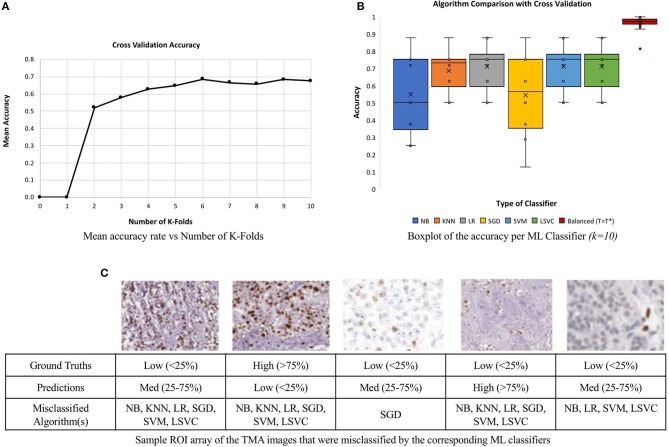
Comparison of six machine learning classifiers to the unsupervised approach on a multi-institutional dataset of 80 Ki67 TMAs. **(A)** Mean classification accuracy of all six classifiers as a function of k in for CV. **(B)** Classification accuracy for each classifier. **(C)** Array of ROI images from misclassified TMAs with labels and the predicted classification.

To minimize issues with small dataset sizes, cross validation (CV) (Efron and Tibshirani, 1997; Saeb et al., 2017; Willis and Riley, 2017) is used. This divides the data into *k* sub-datasets, performs training and testing on each fold and gets average classification performance. In this work, we varied k and learning experiments were conducted, where a single randomized sub-dataset was utilized as the testing data. The accuracy rates of each *k* runs were stored and analyzed. The mean accuracy of each classifier was calculated for a range of *k* values, *k* = *2–10*, similar to Saeb et al. (2017). Figure 12A, illustrates the convergence of the mean accuracy value, after *k* = *6*. In order to select a value of *k*, we must consider bias-variance tradeoffs. According to James et al. (2013) and Kuhn and Johnson (2013) *k* = *5* or *k* = *10* are common values that achieve optimal test errors which while minimizing variance and bias, therefore, the CV method utilized *k* = *10* sub-datasets, which is also supported by the experimental results in Figure 12A (Delen et al., 2005; Polat and Güneş, 2007).

The boxplot, shown in Figure 12B, represents the classification accuracy of each classifier for *k* = *10*, alongside the balanced threshold (*T* = T^*^) for all 80 TMAs. The highest accuracy obtained from the different supervised classification methods in combination with CV was 70.9% and that is for the LR classifier. The NB and SGD classifiers have the lowest classification accuracy and largest variation, indicating lower consistency across the datasets. Although KNN, LR, SGD, SVM, and LSVC have better classification accuracy and are less variable, in general, each classifier has poor to moderate classification performance with wide variance, indicating the classifiers may have trouble generalizing to multicentre data. Figure 12C, illustrates an array of a few of the misclassified ROIs, along with the corresponding ML classifiers and predictions. Notice, each ROI has a different level of biomarker expression, Ki67 staining, as well as different variations of background stromal staining. Compared to the total percentage of correctly classified TMA images using the proposed unsupervised algorithm, the classification accuracy is 92.5%, which is significantly higher than all the supervised methods. Additionally, the variance of the proposed approach is low, demonstrating high reliability and reproducibility of the method.

These results are further summarized in Table 2, which presents the average accuracy rate along with standard deviation for all supervised methods and the proposed, unsupervised method. The standard deviation represents the overall distribution of the results obtained per run (*k* = *10*). The lowest standard deviation achieved by the ML classifiers was 0.11, KNN, which reveals that KNN had the lowest amount of variation and therefore higher reproducibility over the other ML methods.

**Table 2:** Performance comparison to machine learning classifiers.

***K* = 10**	**Gaussian Naïve Bayes**	***K*-Nearest neighbor**	**Logistic regression**	**Stochastic gradient descent**	**Support vector**	**Linear support vector**	Balanced histogram (T = T*)
Accuracy	0.5464	0.6839	0.7089	0.7089	0.7089	0.7089	0.925
Standard deviation	0.207512	0.113796	0.125013	0.158124	0.125013	0.125013	–

The proposed method does not have a standard deviation since CV was not necessary, therefore the framework was only run on the entire dataset once, in comparison to the ML classifiers which were run 10 times. Furthermore, the balanced histogram method achieved the highest results without being computationally expensive and on a minimal dataset, which is beneficial to the consumer. This allows users to utilize the framework without prior training methods, on singular data, and at a faster computation rate. Evidently, the balanced threshold algorithm has benefits in accuracy, not requiring labeled data and is computationally efficient.

## Discussion

A novel color separation methodology was proposed as an alternative method to current stain separation methods in combination with an automatic cell radius estimator. The proposed framework is fully reproducible as it does not require user defined parameters or color vector estimates to achieve stain separation and nuclei detection. The framework can also be applied to entire tissue micro-arrays (TMA) and therefore does not need a region of interest cropped image, hence, minimizing user interface interactions.

The addition of a cell radius estimate to Qi et al. (2012) allowed for a fully objective nuclei detection method that eliminates the requirement for user defined feature selection. This selection in combination with the color separated images resulted in a stain specific cell radius estimate. Since tumor cells are often associated with larger morphological characteristics, the selection of a unique radius estimate generates a more accurate nuclei detection procedure, opposed to assuming a single radius for all cells in an image. The adaptive cell radius estimator was able to achieve a comparable result to arbitrarily selected parameters. Although performance was slightly lower than the ideal estimates, reliance on a potentially inaccurate user defined parameter increases the risk of unreliable and subjective results. Automatic cell radius generation ensures a more robust analysis, however, identification of smaller or elongated nuclei remains a challenge for this detection technique. Nuclear boundaries are not considered for this work as identification would greatly increase computational requirements and nuclear boundaries do not contribute to nuclei count based PI calculations.

Color separated images were created to model stain concentrations in Ki67 and hematoxylin stained images. The b^*^ color channel from the L^*^a^*^b^*^ color space was able to effectively differentiate stain content in a way that mimics the HVS perception of color content. An important feature of the color separation method is that the range of color intensities observed in the b^*^ channel define the maximum values observed in the color separated images. By normalizing the confidence images to the maximum staining intensity observed in the current image, the proposed algorithm is robust to staining variations common to histopathology images. This is especially important for Ki67 staining because positive Ki67 identification is not dependent on the overall amount of staining intensity. For images with low stain representations, an object with very light Ki67 staining may be considered positive by a pathologist opposed to an image containing high Ki67 content where the same light staining may be associated with stromal or other background content.

The proposed algorithm has the ability to robustly analyze various types of staining, i.e. over/under staining, by adaptively selecting a threshold that compensates accordingly. However, the although color content may be the most clearly identifiable factor in IHC analysis, the algorithm does not examine additional factors a pathologist may consider when performing nuclei classification. Additionally, due to stain mixing some nuclei containing both Ki67 and hematoxylin result in grayscale content, leading to low representation of the color intensity images.

The outlier image, in the OVC dataset, contained very high levels of Ki67 stain content characteristics of over staining, making it difficult to identify healthy and abnormal nuclei without additional considerations such as cell morphology. The outlier image's ground truth PI value was directly compared to the proposed method, natural threshold, Otsu method, CD methods, and ML algorithms' obtained PI quantification. Of the comparing methods, the proposed algorithm achieved the lowest PI difference. Despite these challenges, the proposed color separation technique was able to attain accurate PI estimates for all images that have a variety of PI values. Combining the OVC and Protein Atlas datasets, a total of 80 TMA images, the proposed algorithm was able to achieve an overall accuracy rate of 92.5%.

**Figure 13:**
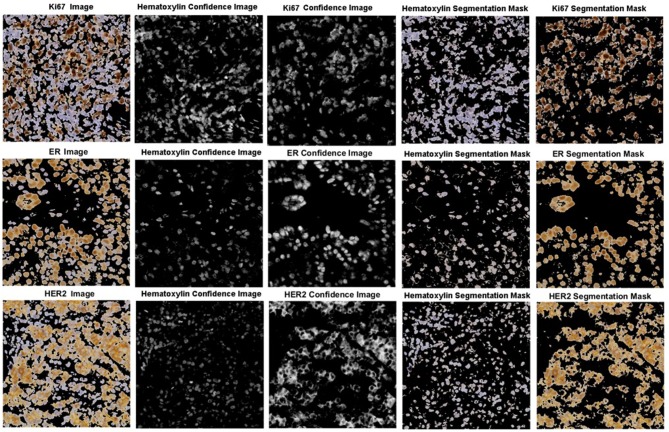
Color separation framework applied to Ki67, ER, and HER2 images with associated confidence and color separated images.

The robustness of the color separation also allows for potential implementation for other IHC stains. The proposed color separation method is shown for ER and Her2 stained images, as seen in Figure 13. These images are from different scanners, which contain different staining intensities, concentrations, and utilize different biomarkers. Despite these differences, the color separation framework is able to effectively separate the hematoxylin content from the respective IHC stain.

Future works on improving this framework include adapting a fuzzy membership and a moving window methodology, into the color thresholding algorithm, alongside an interactive GUI for pathologists' optional technical input. Implementation of the proposed algorithm in clinical practice would limit the subjectivity within standard scoring systems, help define robust cut-off thresholds for patient treatment options, and assist the pathologist in forming a complete and robust prognosis. Overall, the proposed framework would improve patient quality of care by allowing pathologists to accurately complete prognosis for every patient within minimal time.

## Conclusion

The proposed unsupervised Ki67 PI calculator provides robust and accurate results with multi-institutional TMA core images. The framework accurately analyzed 80 TMA Ki67 stained images, from two contrasting continental laboratories and three different whole-slide scanners with variable image magnification. The images consisted of wide ranges of color variability due to various scanning hardware, compounds, vendors, tissues, and different levels of biomarker expression. An average proliferation index difference of 3.25% from 30 OVC TMAs and an accuracy rate of 90% for 50 Protein Atlas TMAs was achieved. As a whole, the total framework achieved an accuracy rate of 92.5%, over 80 TMA images, which outperformed six machine-learning algorithms and two popular color deconvolution (CD)-based Ki67 PI quantification methods. The proposed framework is an effective measure of proliferation activity that is robust to stain variation between images.

## Data Availability Statement

The publicly available dataset that was analyzed in this study can be found here: https://www.proteinatlas.org/ENSG00000148773-MKI67/pathology/breast+cancer#.

## Ethics Statement

Biopsies from the Ontario Veterinary College, University of Guelph, provided to the IAMLAB. The dataset was retrospective data, hence no consent was required.

## Author Contributions

Overall contribution to write and edit the manuscript was as follows, AK and RG contributed 25%, PM attributed 20%, DA and EB supported 10%, and 5% from GW and RD.

### Conflict of Interest

The authors declare that the research was conducted in the absence of any commercial or financial relationships that could be construed as a potential conflict of interest.
